# Fluorescent porous organic polymers for detection and adsorption of nitroaromatic compounds

**DOI:** 10.1038/s41598-022-20024-x

**Published:** 2022-09-23

**Authors:** Jia-Bin Xiong, Ding-Ding Ban, Yong-Juan Zhou, Hui-Jun Du, Ai-Wei Zhao, Lan-Ge Xie, Guo-Qun Liu, Si-Ru Chen, Li-Wei Mi

**Affiliations:** 1 School of Material and Chemical Engineering, Center for Advanced Materials Research, Zhongyuan University of Technology, Zhengzhou, 450007 People’s Republic of China; 2 College of Chemistry, Green Catalysis Center, International Phosphorus Laboratory, International Joint Research Laboratory for Functional Organophosphorus Materials of Henan Province, Zhengzhou University, Zhengzhou, 450001 People’s Republic of China

**Keywords:** Chemistry, Materials science

## Abstract

A fluorescent porous organic polymer (FPOP) with strong fluorescence and tunable emission colors, was synthesized through a simple cost-effective method via Scholl coupling reaction. Experiments proved the stability and excellent detection and adsorption ability, and microporous nature of the material. Luminescence of FPOP was quenched when addition of nitroaromatic compounds. The properties along with large-scale and low-cost preparation make these FPOP potential candidates for fluorescence detection of nitroaromatic compounds. Additionally, FPOP shows higher adsorption capacity and rate than other reported adsorbents, and has the possibility of being an effective adsorbent for industrial usage. Moreover, a fluorescent test paper was further developed and is found to be sensitive to 10^–8^ M level, complete with a rapid response time and visual detection. This newly developed strategy may open up an avenue for exploring porous polymers, particularly those with a strong fluorescence, for the large-scale fabrication of FPOP for various advanced applications.

## Introduction

Porous organic polymers constructed by covalently linked porous materials exhibit unique structural and functional diversity and possess the advantages of a high specific surface area, low cost, ease modifiability, chemical robustness (e.g., acids and alkaline resistance, organic solvent tolerance, and so on), which have been developed rapidly in the area of gas adsorption^1–3^ and separation^4–6^, catalysis^7–9^, energy storage^10–12^, sensing^13–15^, etc. The porous organic polymers mainly include: hyper-crosslinked polymers^16^, conjugated microporous polymers^17^, porous aromatic framework^18^, covalent triazine framework^19^, and covalent organic frameworks^20^. Furthermore, it should be emphasized that a variety of functional porous organic polymers have been prepared by introduction different moieties, such as fluorophore, recognitive active site, or chemical group, with potential functional properties^21–23^.

Research interest on detection and adsorption of nitroaromatic compounds has exhibited considerable increase in recent years^24,25^. While some instrumental techniques have been utilized for sensing of nitroaromatic compounds, such as fluorescence spectrometry and atomic adsorption spectrometry, complicated detection process (sample preparation and equipment operation) are generally required^26^. Comparatively, fluorescence-based methods have several advantages for the detection of nitroaromatic compounds, such as relatively simplicity, high sensitivity and selectivity^27^. For luminescent sensors, the reported work mainly includes: small organic molecules, metal–organic complexes, crystalline porous materials, etc^25,28,29^. But most of these materials lack chemical and thermal stability over a wider range, especially for industrial applications, and difficult to prepare on a larger scale. Fluorescent porous organic polymer (FPOP) constructed by hyper-crosslinked polymers, providing considerable opportunities for their promising application in the area of detection and adsorption of nitroaromatic compounds. On the one hand, the introduced microporous structures possess high specific surface area which can greatly improves the contact between the fluorescent porous structures and the target analytes^30^; On the other hand, the synthetic process is simple and cost-effective for the preparation of FPOP materials^31,32^. However, hyper-crosslinked polymer based porous organic polymers, which are prepared cost-effective and show rapid and excellent fluorescence detection ability for nitroaromatic compounds, still less unexplored. Moreover, although numerous fluorescent porous organic polymers have been synthesized from different functional building blocks, well-defined adsorbent effectively works for the same type of pollutant (e.g., containing benzene or electron withdrawing group) remain rather limited.

In this work, we constructed a series of fluorescent porous organic polymers containing different fluorophore moieties (tetraphenylethene, pyrene, and spirobifluorene) and exhibiting varied fluorescence colors by Scholl coupling reaction. The preparation process is simple and cost-effective, exhibiting chemical and thermal stability. Furthermore, the sensitivity and selectivity of hyper-crosslinked porous organic polymers with fluorescence characteristics for detecting nitroaromatic compounds have been explored, which exhibited efficiently high quenching rate and adsorption amount. More importantly, they exhibit a strong fluorescence and tunable emission colors, the developed rapid response time and visual fluorescent test paper was sensitive to 10^–8^ M level.

## Results and discussion

### Synthesis and structural characterization

**Figure 1:**
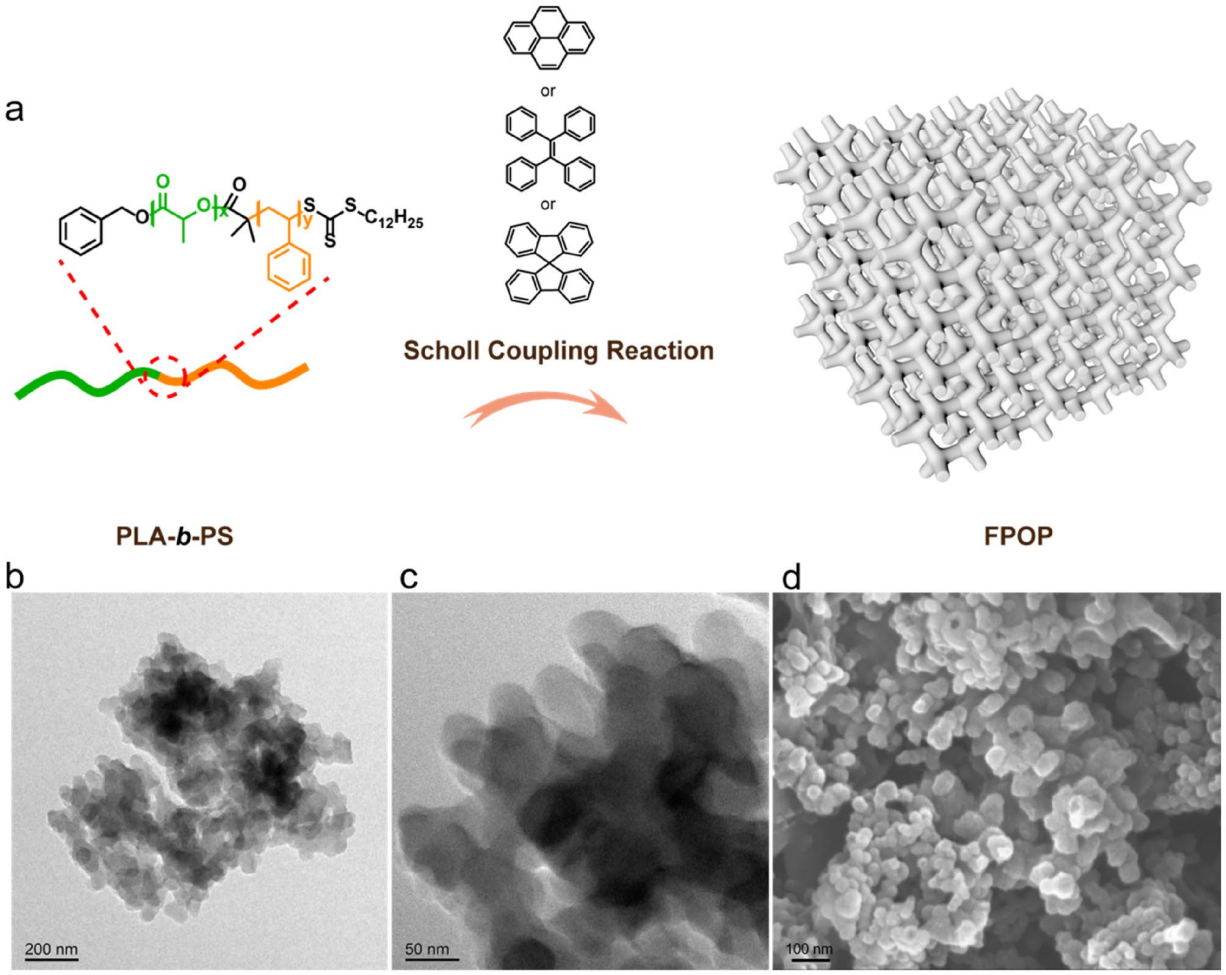
Synthesis and characterization of FPOP-TPE. (**a**) Schematic illustration of the preparation of FPOP-TPE. (**b, c**) Typical TEM images in a different magnification of FPOP-TPE. (**d**) SEM image of FPOP-TPE.

The precursors of polylactide-*b*-polystyrene (PLA-*b*-PS) were firstly synthesized according to the previously reported method^33^. Figure 1a illustrates the synthesis procedure for introduce a typical luminescent unit (tetraphenylethene (TPE), pyrene, and spirobifluorene) into the porous organic polymers by Scholl coupling reaction of PLA-*b*-PS precursor with the luminescent unit. The obtained fluorescent porous organic polymers denote as FPOP-TPE, FPOP-Py, and FPOP-Sp, respectively. As a comparison, the Scholl coupling reaction of TPE, pyrene, and spirobifluorene without PLA-*b*-PS precursor were also synthesized (see the Supporting Information for details). These FPOP exhibit similar absorbance behavior, i.e., strong and broad with the maximum peaks at ~ 350 nm in the UV–Vis absorption spectra (Figure S1). The FPOPs all exhibit a strong fluorescence with emission colors from brick-red to yellow and blue under UV light (Figure S2). Here, we chose FPOP-TPE to characterize the structure in detail because they exhibit similar physical properties. The morphology of FPOP-TPE was characterized by transmission electron microscopy (TEM) and scanning electron microscopy (SEM). TEM analysis shows the amorphous structure (Fig. 1b, c), and SEM image (Fig. 1d) further shown the outline somewhat similar to the blocky structure. The absolute fluorescence quantum yield (ϕ) was determined by Wrighton–Ginley–Morese’s method to be 14.47% for FPOP-TPE (Figure S3). The structure of FPOP-TPE was determined by FT-IR, solid-state ^13^C CP/MAS NMR, and elemental analysis (Figures S4–S5).

**Figure 2:**
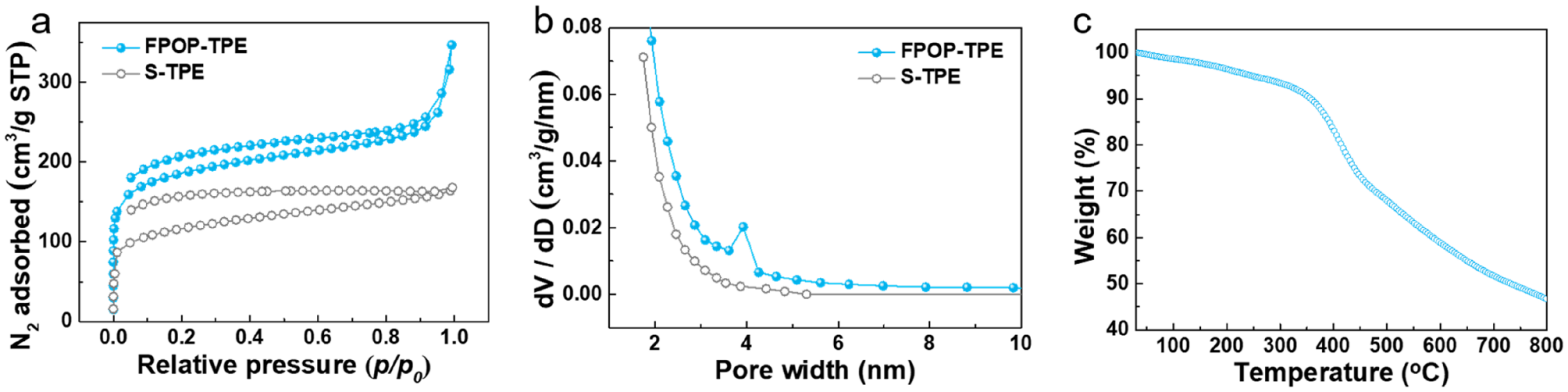
(**a**) N_2_ adsorption–desorption isotherms, and (**b**) pore size distribution of FPOP-TPE and S-TPE, calculated from the adsorption branch of isotherms using the NLDFT model. (**c**) TGA curve of FPOP-TPE under nitrogen atmosphere.

N_2_ adsorption isotherm measurements were carried out at 77 K to measure the surface area and porosity of the resultant FPOP-TPE and S-TPE (Scholl coupling reaction of TPE monomer, denote as S-TPE, Figure S6). As shown in Fig. 2a, the isotherm of FPOP-TPE exhibit a typical type I and type IV isotherm with steep increase at low relative pressure, and S-TPE exhibit a typical type I isotherm, indicating the existence of microporous structure. The corresponding pore size distribution of FPOP-TPE with pore sizes of *ca*. 4 nm and S-TPE with pore sizes mainly less than 2 nm, based on the nonlocalized density functional theory (NLDFT) (Fig. 2b). Brunauer–Emmett–Teller (BET) surface area (Table S1) also provides the detailed specific surface area and pore volume data. Thermogravimetric analysis (TGA) was performed to determine the thermal stability of the FPOP-TPE. It can be found that FPOP-TPE is thermally stable even at 400 °C (Fig. 2c).

### Adsorption and detection of nitroaromatic compounds

**Figure 3:**
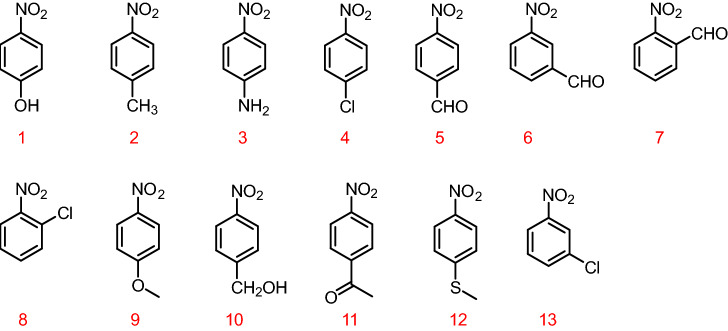
Molecule structure of different nitroaromatic compounds.

**Figure 4:**
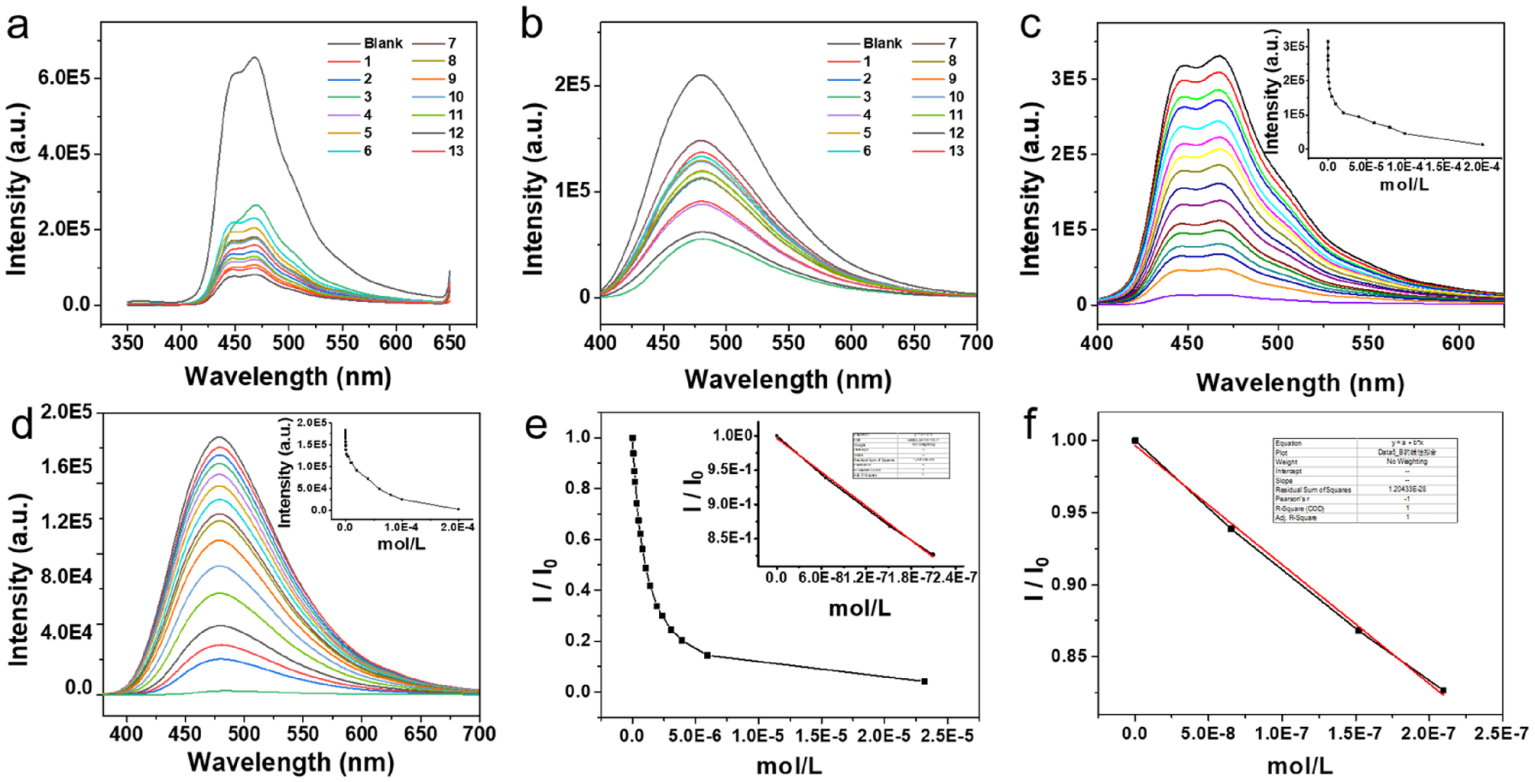
Fluorescence spectra [FPOP-SP] = 3 mg/mL (Ethanol/Water = 90/10) [Nitroaromatic compounds] = 2 × 10^–5^ M. (**a**) ex = 330 nm, ex/em slits = 5/10 nm. (**b**) ex = 365 nm, ex/em slits = 5/5 nm. (**c**) ex = 330 nm, slits = 5/10 nm. (**d**) ex = 365 nm, ex/em slits = 5/5 nm (**e**) I and I_0_ represent the fluorescence intensities of the suspension with and without nitroaromatic compounds added at a certain ratio. ex = 330 nm, slits = 5/10 nm. (**f**) Take data between 0–2 × 10^–7^ linear fit was performed; ex = 330 nm, slits = 5/10 nm.

Since the designed and synthesized FPOP contains a large number of electron-rich phenoxy groups, the doped fluorophore with a large conjugated structure makes the overall polymer rich in electrons. Therefore, there will be a strong interaction between FPOP and electron-deficient nitroaromatic compounds. Theoretically, when FPOP-Sp and FPOP-TPE interact with nitroaromatic compounds Nos. 1 to 13 (Fig. 3), respectively, the fluorescence will decrease to different degrees. As shown in Fig. 4a, b, apparent fluorescence quenching is observed when FPOP-Sp encounters nitroaromatic compound 12 and FPOP-TPE encounters nitroaromatic compound 3, which may be more compatible with the structural channel and small molecule, and the interaction distance is closer, the effect is better. Next, the research plan is to carry out molecular simulation, calculate the action site and energy, and compare it with the experimental results to see the accuracy of the main conjecture.

According to the above screening results, fluorescence titration experiments were performed on FPOP-Py and FPOP-Sp respectively. The results are shown in Fig. 4c, d. After the host compound was dispersed into the solution, the nitroaromatic compound was quantitatively added. The orderly and gradual quenching of fluorescence can confirm that the interaction between the host and the guest causes fluorescence quenching, rather than an accidental phenomenon. Comparing the titration results of the two compounds, it was found that the detection limit was calculated by the concentration corresponding to the triple error, or the concentration corresponding to the 5% signal change. The minimum detection concentration of FPOP-Sp is 2 × 10^–8^ mol/L, and the minimum detection concentration of FPOP-TPE is 5 × 10^–8^ mol/L, indicating that the sensitivity of FPOP-Sp is high, and this result is also proved again in the fluorescence test paper experiment.

In Fig. 4e, according to the Stern–Volmer curve made, it is quenched in exponential form, and such quenching types can be both dynamic quenching and static quenching. The linear fitting of the first four titration points of the initial concentration can reach a variance of 0.999, which indicates that the titration results have high accuracy and good data reproducibility. The same experiment was also performed for FPOP-TPE (Figure S7). The sensitivity of FPOP-TPE is not as high as that of FPOP-Sp. If you take the first point, you will not get a better variance number. In addition, solid-state fluorescence was also tested (Figure S8). Both FPOP-SP and FPOP-TPE have better luminescence in solid state, which are more favorable for application as fluorescent materials. To investigate the reusability of the FPOPs, five cycles of over FPOP-Py and FPOP-Sp were then performed. As shown in Figures S9–S10, after five cycles, there were no obvious changes of fluorescence decay. These results indicate that the FPOP-Py and FPOP-Sp possesses excellent reusability.

After the identification of the nitroaromatic compound of the material, the adsorption experiment was tested. First, make a UV standard curve (Figure S11), and test the absorption value of nitroaromatic compound 3 at different concentrations. The calculated variance is 0.993 (Figure S12), which can be effectively compared. Stir and adsorb the porous material in the nitroaromatic compound solution, filter and wash the surface, stir in pure solvent to desorb the adsorption, absorb the supernatant for UV absorption value test (Figure S13), and compare the standard curve to calculate the adsorption value. The results are shown in the Table S2, each milligram of porous material can adsorb more than 0.7 mg of nitroaromatic compounds, which has a large adsorption capacity, which provides the possibility for practical application and industrial-grade adsorption.

**Figure 5:**
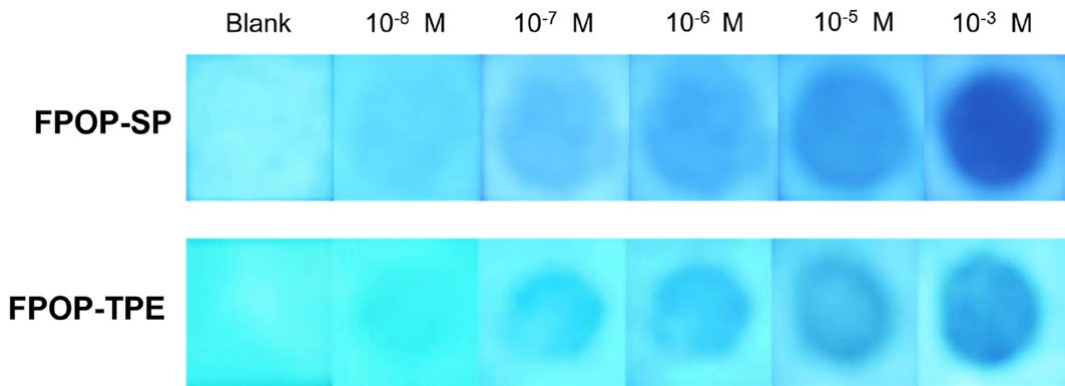
After the test paper containing FPOP-SP and FPOP-TPE compounds was spotted with different concentrations of nitroaromatic compounds 3, pictures were taken under 365 nm UV light.

In the application of fluorescent probes, in addition to high selectivity and sensitivity, in the actual environmental test, it needs to have the characteristics of simplicity and speed. In order to achieve this purpose, we designed and prepared fluorescent test strips for nitroaromatic compounds, and took pictures for the test, and obtained good results (Fig. 5). The preparation process is simple. The laboratory filter paper is cut into a (1.0 × 1.0 cm) size square, and the main compound is dispersed with ethanol to obtain the suspensions of FPOP-SP and FPOP-TPE, respectively. Put the square paper into the suspension for infiltration. Soak it, take it out and dry it at 60 °C for later use, and then a fluorescent test paper for quick and simple inspection of nitroaromatic compounds can be obtained. During the test, at the center of the test paper, carefully spot 10 µL ethanol solutions of nitroaromatic compound 3 with different concentrations, and a dot of about 0.5 cm^2^ can be obtained. After the dots are dried, they are irradiated with a UV lamp immediately, and the fluorescent quenched dots can be observed. The test results are in good agreement with the prediction. As the concentration of nitroaromatic compounds increases, the fluorescence intensity of the central dot of the fluorescent test paper gradually decreases. The lowest concentration of nitroaromatic compounds that can be observed by the naked eye is about 1 × 10^–8^ M level, which can meet the needs of most routine tests.

## Conclusion

In summary, a class of highly fluorescent porous organic polymers with tunable emission wavelengths was simply and efficiently synthesized by the Scholl coupling reaction. Through research, it is found that this kind of porous organic polymer can effectively detect nitroaromatic compounds and has good adsorption capacity. It has good advantages in terms of synthesis economy, rapid detection, and large-scale adsorption. The effective detection concentration of the polymer for nitroaromatic compounds can reach 10^–8^ M, and at this concentration, the prepared fluorescent test paper can also be discerned by the naked eye. The adsorption results show that this kind of polymer can effectively adsorb 0.7 mg of nitroaromatic compounds per mg, and has a good adsorption capacity. Such materials can be used as promising ideal adsorption materials for the identification and adsorption of nitroaromatic compounds in practical environments, and can be used as a class of industrial-grade materials that can effectively adsorb and remove trace amounts of nitroaromatic compounds in large systems.

## Experimental details

### Materials

All the commercially available chemical reagents were supplied by Adamas Reagent Ltd., 3A Chemicals, Shanghai Macklin Biochemical Co., Ltd., Shanghai Aladdin Biochemical Technology Co., Ltd., J&K, Sinopharm (China). Other analytical grade solvents and reagents were used without further purification.

### Synthesis of fluorescent porous organic polymer

Anhydrous AlCl_3_ (4 eq., 160 mg) was added to a solution of PLA-*b*-PS (1 eq., 100 mg) and TPE (1 eq.) in CHCl_3_ (10 mL) with vigorous stirring at 58 °C, the reaction proceeded for 1 h. The solid product FPOP-TPE was washed twice with methanol, twice with HCl/H_2_O (v:v = 1:5), and then three times with methanol. Followed by ethanolic Soxhlet extraction with ethanol for 48 h, prior to drying in a vacuum oven at room temperature for 24 h. The polymer was obtained as a light grey solid. The other fluorescent porous organic polymers (FPOP-Py, FPOP-Sp) were synthesized with the same method, except for using pyrene and spirobifluorene to replace TPE. The yields of FPOP-TPE, FPOP-Py, and FPOP-Sp is 76%, 80%, and 78%, respectively. Elemental analysis for FPOP-TPE: C 83.2%, H 5.6%.

The Scholl coupling reaction of TPE, pyrene, and spirobifluorene were synthesized similar to the FPOP. For example, Scholl coupling reaction of TPE (denote as S-TPE): anhydrous AlCl_3_ was added to a solution of TPE in CHCl_3_ with vigorous stirring at 58 °C, the reaction proceeded for 1 h. The solid product S-TPE was washed twice with methanol, twice with HCl/H_2_O (v:v = 1:5), and then three times with methanol. Followed by ethanolic Soxhlet extraction with ethanol for 48 h, prior to drying in a vacuum oven at room temperature for 24 h. The polymer was obtained as a dark brown solid. The other Scholl coupling reaction of pyrene and spirobifluorene were synthesized with the same method, except for using pyrene and spirobifluorene to replace TPE, we denote them as S-Py and S-Sp, respectively. The yields of S-TPE, S-Py, and S-Sp is 82%, 86%, and 87%, respectively.

## Supplementary Information

Supplementary Information.

## Data Availability

All data generated or analyzed during this study are included in this published article [and its supplementary information files].
