# Supplementary material for: Fluorescent porous organic polymers for detection and adsorption of nitroaromatic compounds
Source: Sci Rep. 2022 Sep 23;12:15876. doi: 10.1038/s41598-022-20024-x (PMC9508238; doi:10.1038/s41598-022-20024-x)
Supplement: Supplementary file 1 — Supplementary Information. [file 41598_2022_20024_MOESM1_ESM.docx]

Supporting Information

Fluorescent Porous Organic Polymers for Detection and Adsorption of Nitroaromatic Compounds

Jia-Bin Xiong^a,b,*^, Ding-Ding Ban^a^, Yong-Juan Zhou^a^, Hui-Jun Du^a^ Ai-Wei Zhao^a^,Lan-Ge Xie^a^,Guo-Qun Liu^a*^ Si-Ru Chen^a*^and Li-Wei Mi^a*^

1. School of Material and Chemical Engineering, Center for Advanced Materials Research, Zhongyuan University of Technology, Zhengzhou 450007, China. E-mail: xjiabin@foxmail.com, mlwzzu@163.com.
2. College of Chemistry, Green Catalysis Center, International Phosphorus Laboratory, International Joint Research Laboratory for Functional Organophosphorus Materials of Henan Province, Zhengzhou University, Zhengzhou 450001, People’s Republic of China.

**Table of Contents**

| **1. Supplementary Figures** | **S3** |
| --- | --- |
| **2. Supplementary Tables** | **S9** |
| **3. Supplementary Methods** | **S10** |
| **4. Supplementary References** | **S11** |

**1. Supplementary Figures**


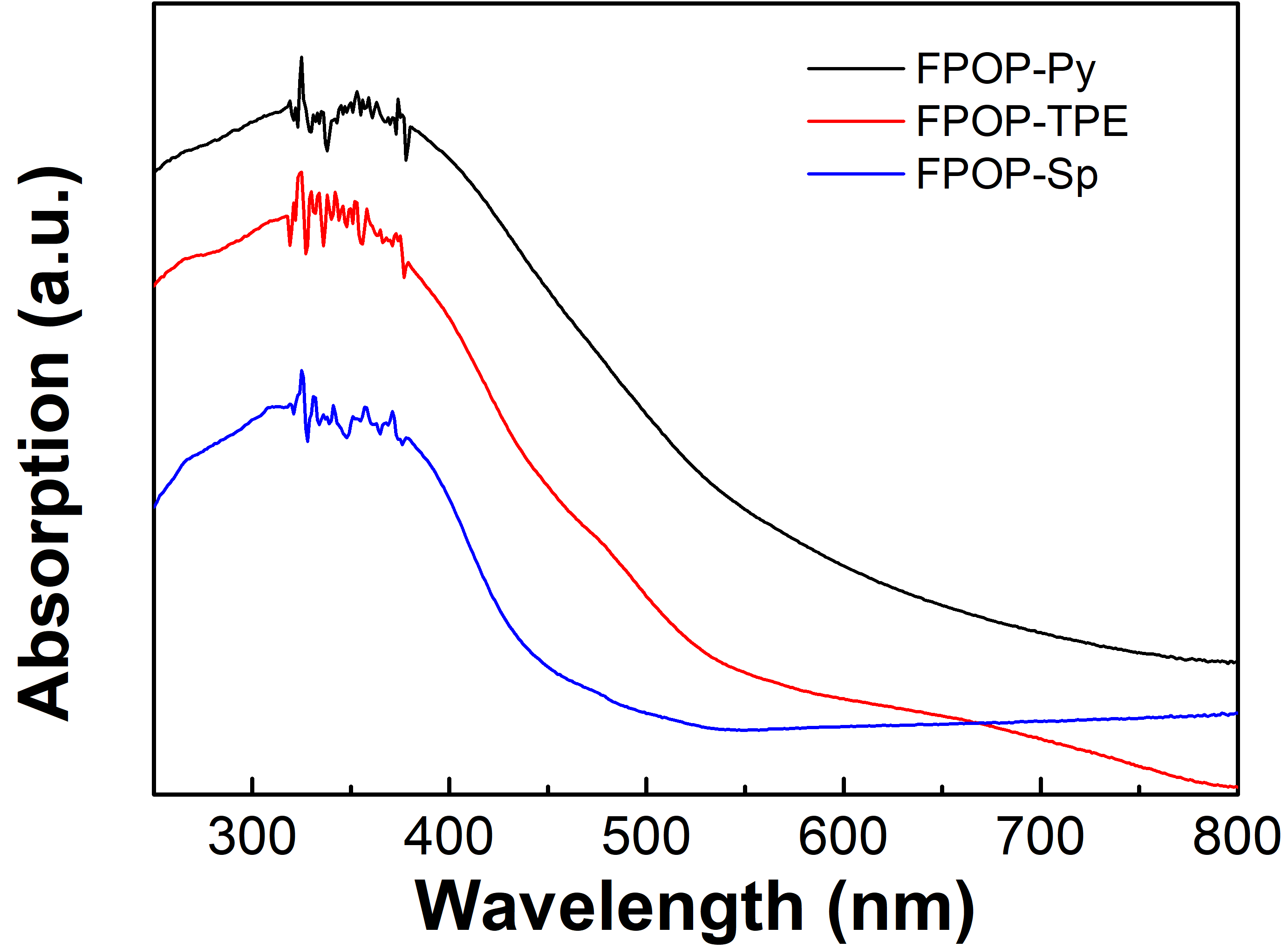


Figure S1. UV-Vis absorption spectra of FPOP-Py, FPOP-TPE, and FPOP-Sp in the solid-state.


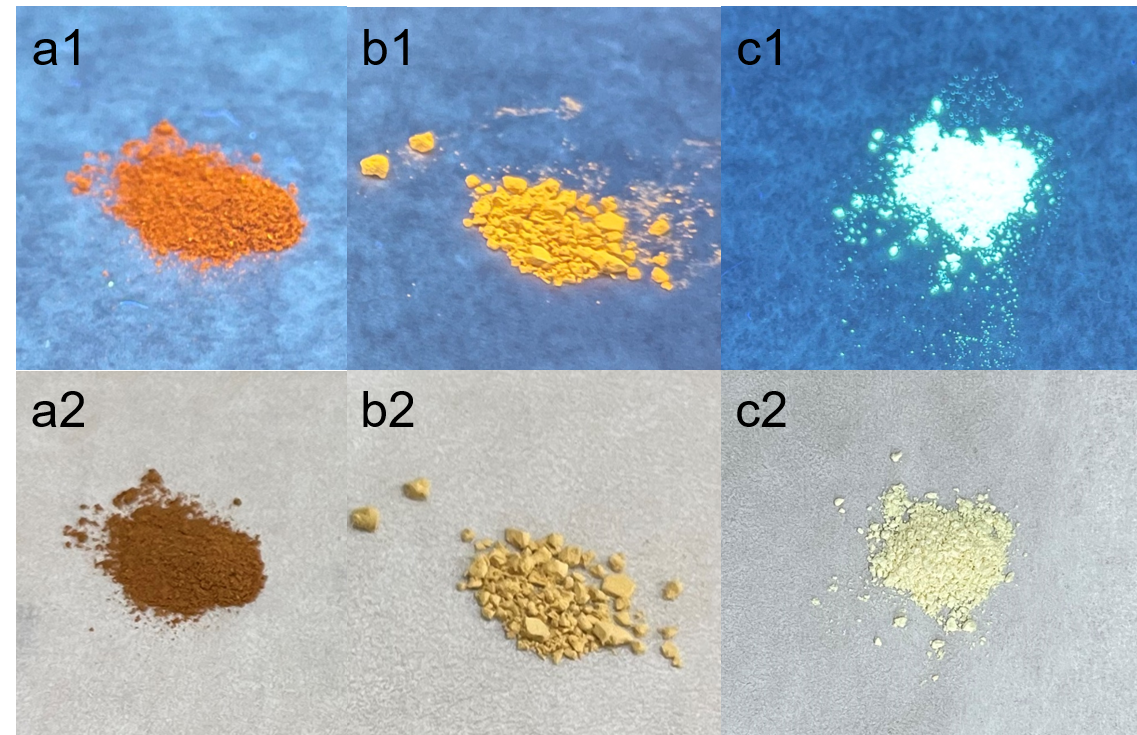


Figure S2. The photographs of FPOP-Py (a1-a2), FPOP-TPE (b1-b2), and FPOP-Sp (c1-c2) under UV light (*λ*_ex_ = 365 nm, upside) and daylight (downside).


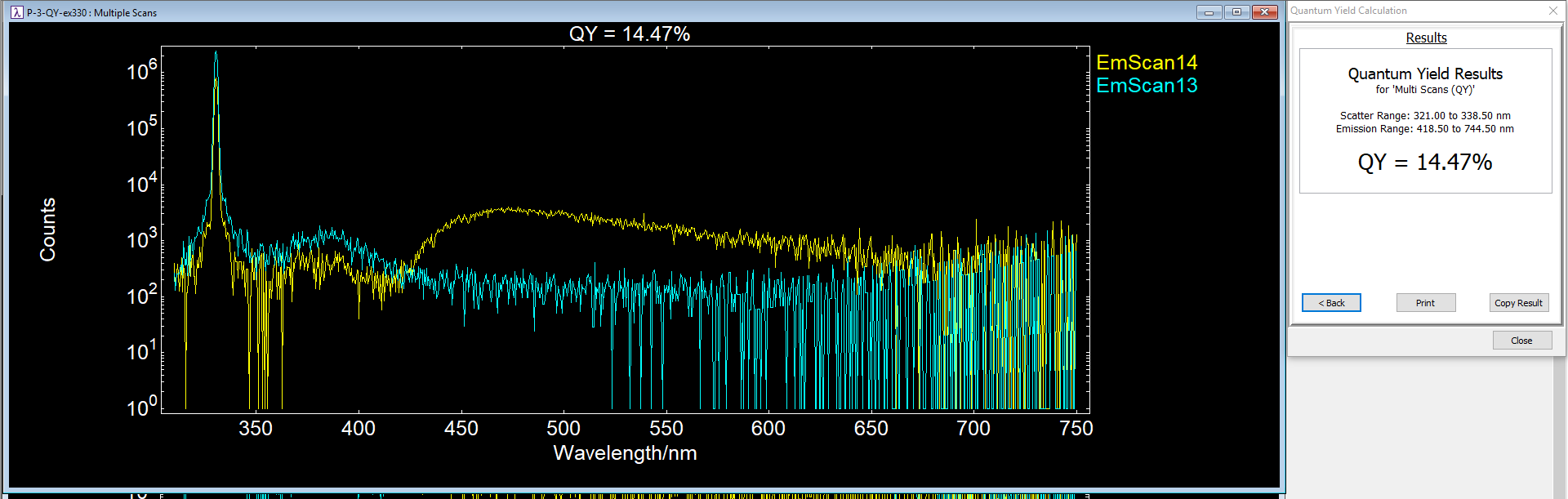


Figure S3. Quantum yield of FPOP-TPE.


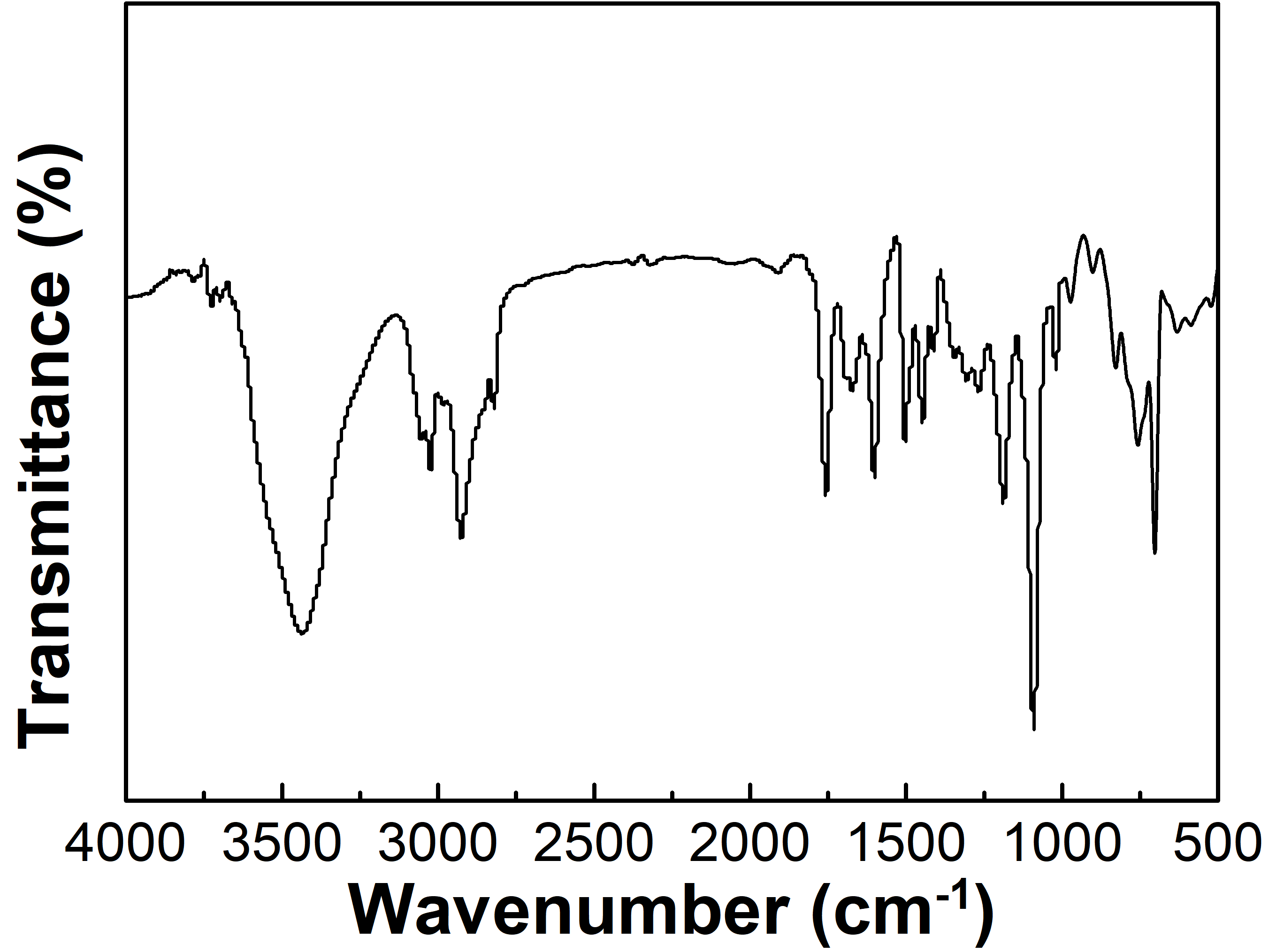


Figure S4. FT-IR spectra of FPOP-TPE. The broad bands at 3600-3100 cm^-1^ are largely attributed to adsorbed water in the networks created. The bands at 3100-2800 cm^-1^ are the C-H stretching vibrations of the benzene ring, while the bands at 1503 cm^-1^, 1447 cm^-1^, and 1411 cm^-1^ are assigned to C=C stretching vibrations of the benzene ring. The characteristic peaks from 1670 to 1410 cm^-1^ are assigned to the C=C stretching vibrations from the vinyl and phenyl groups.


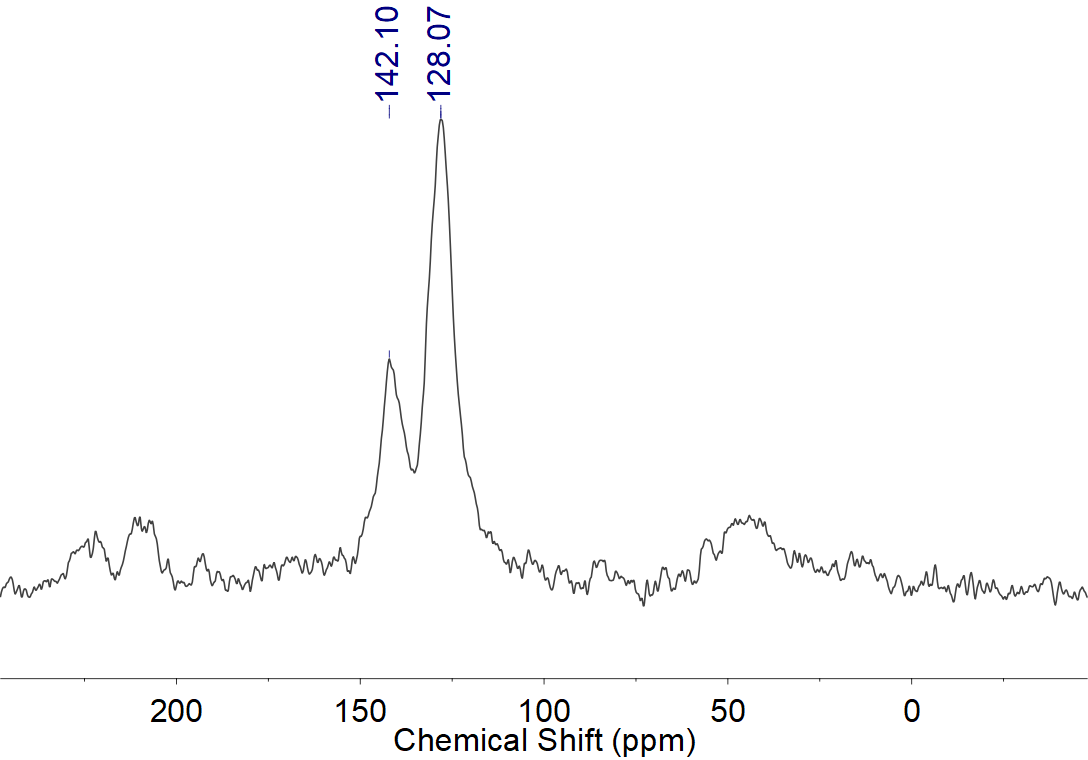


Figure S5. Solid-state ^13^C cross-polarization magic-angle spinning (CP/MAS) NMR of FPOP-TPE. The resonances at ~128 and 142 ppm are respectively due to the non-coupled and coupled aromatic carbons.^1-2^


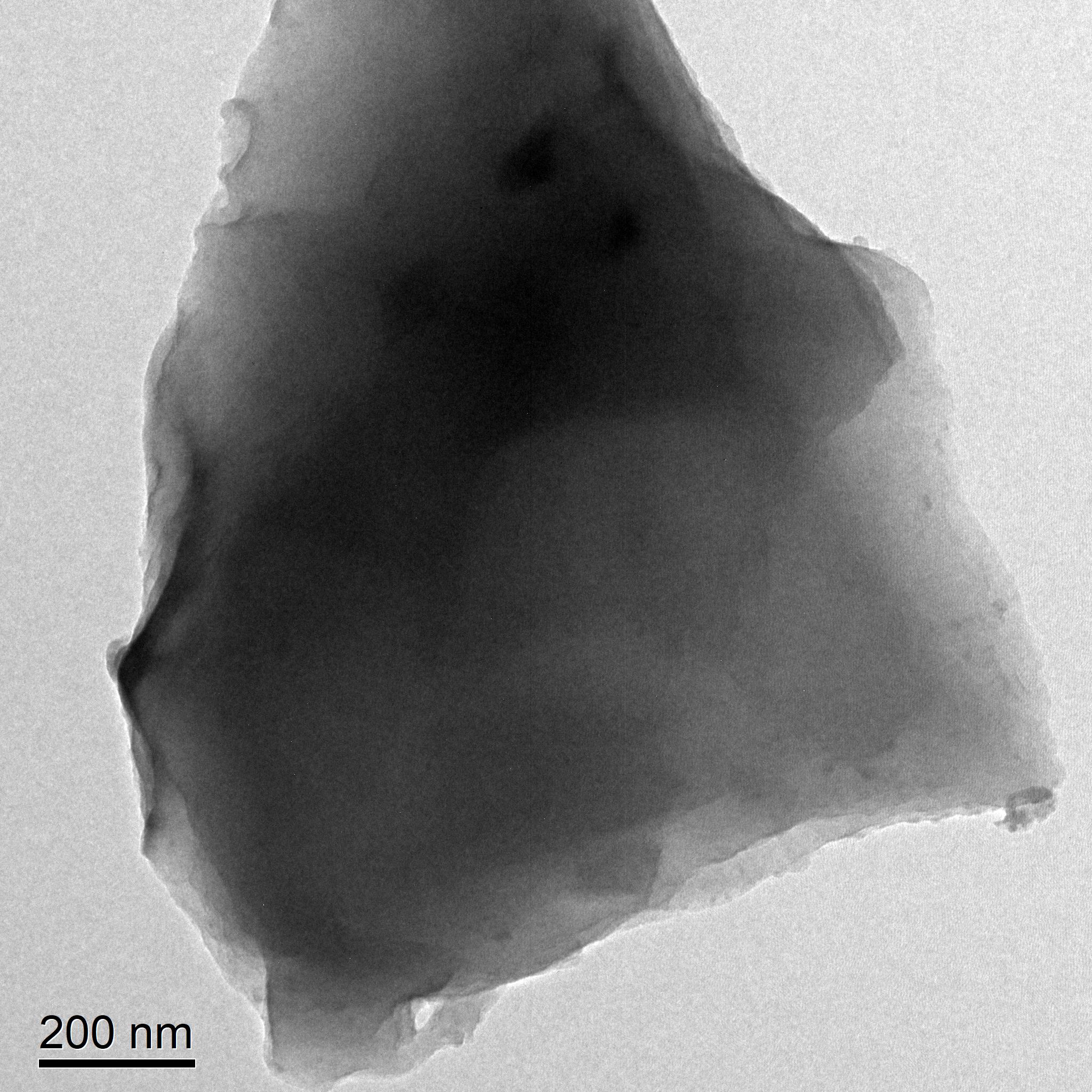


Figure S6. Typical TEM image of S-TPE.

Figure S7. The I/I_0_ value of the suspension varies with the fluorescence of the nitroaromatic compound added; I and I_0_ represent the fluorescence intensities of the suspension with and without nitroaromatic compounds added at a certain ratio. [FPOP-TPE] = 3 mg/mL, ex = 365 nm, ex/em slits = 5/5 nm.

Figure S8. Solid Fluorescence Intensity, ex = 345 nm, ex/em slits = 3/3 nm.


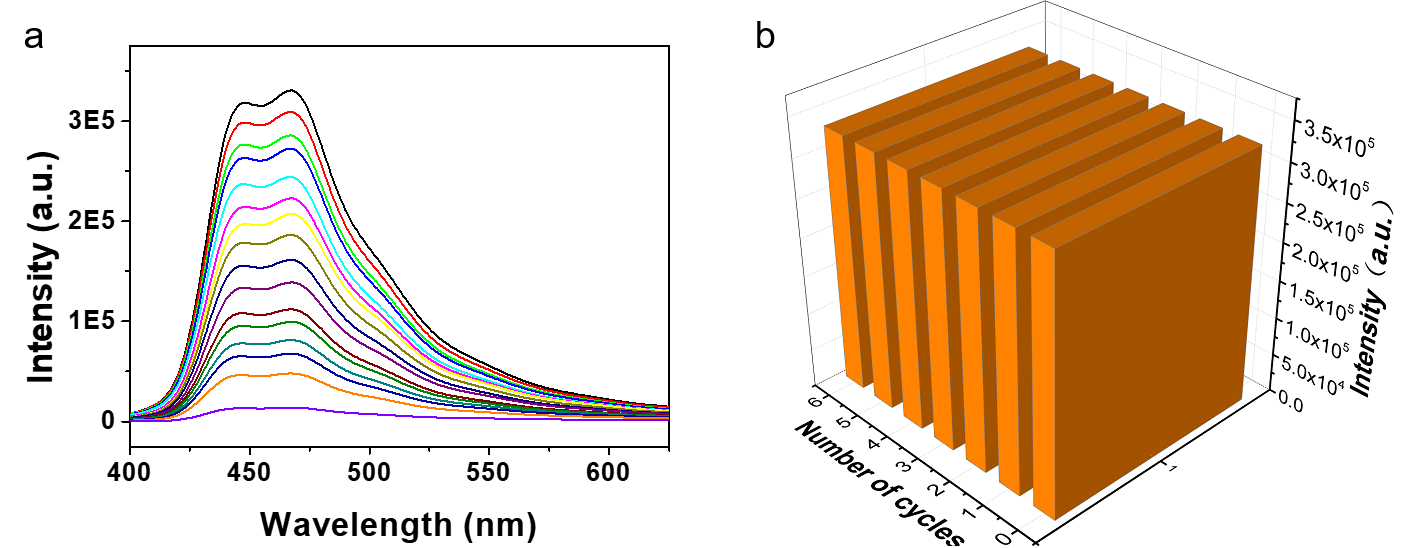


Figure S9. (a) Fluorescence spectra of host compound suspensions when different nitro compounds were added. (Ethanol/Water=90/10), FPOP-Py = 3mg/mL. ex = 330 nm, slits = 5/10 nm. (b) Recycling tests for the reusability of FPOP-Py.


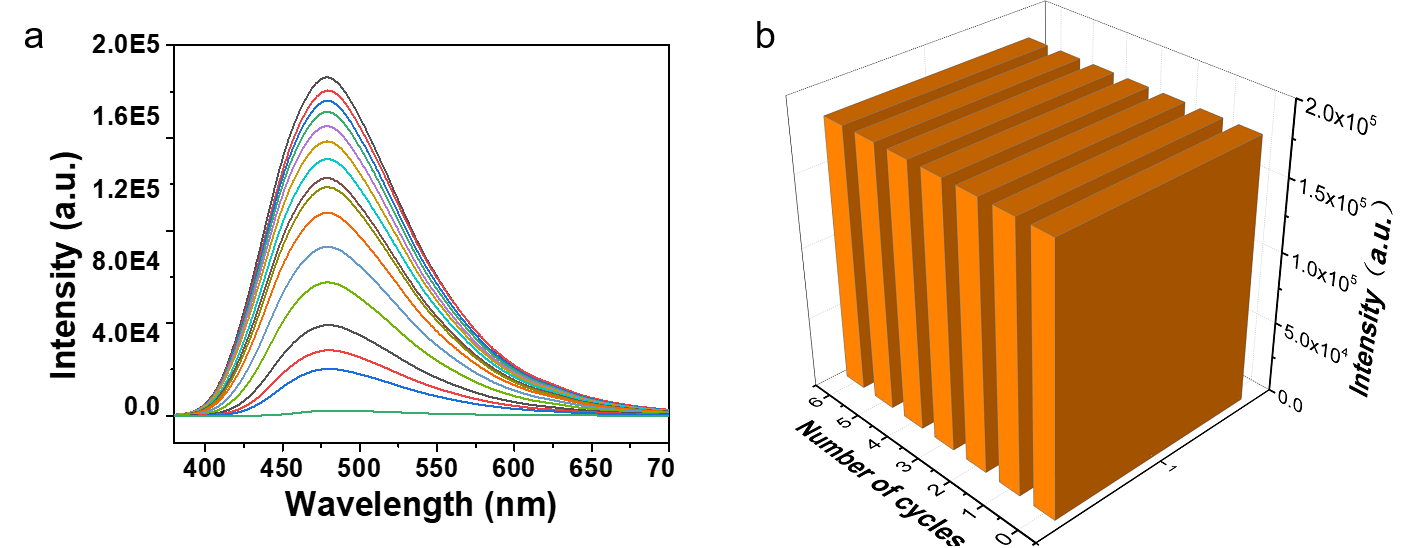


Figure S10. (a) Fluorescence spectra of host compound suspensions when different nitro compounds were added. (Ethanol/Water=90/10), FPOP-Sp = 3mg/mL. ex = 365 nm, slits = 5/5 nm. (b) Recycling tests for the reusability of FPOP-Sp.


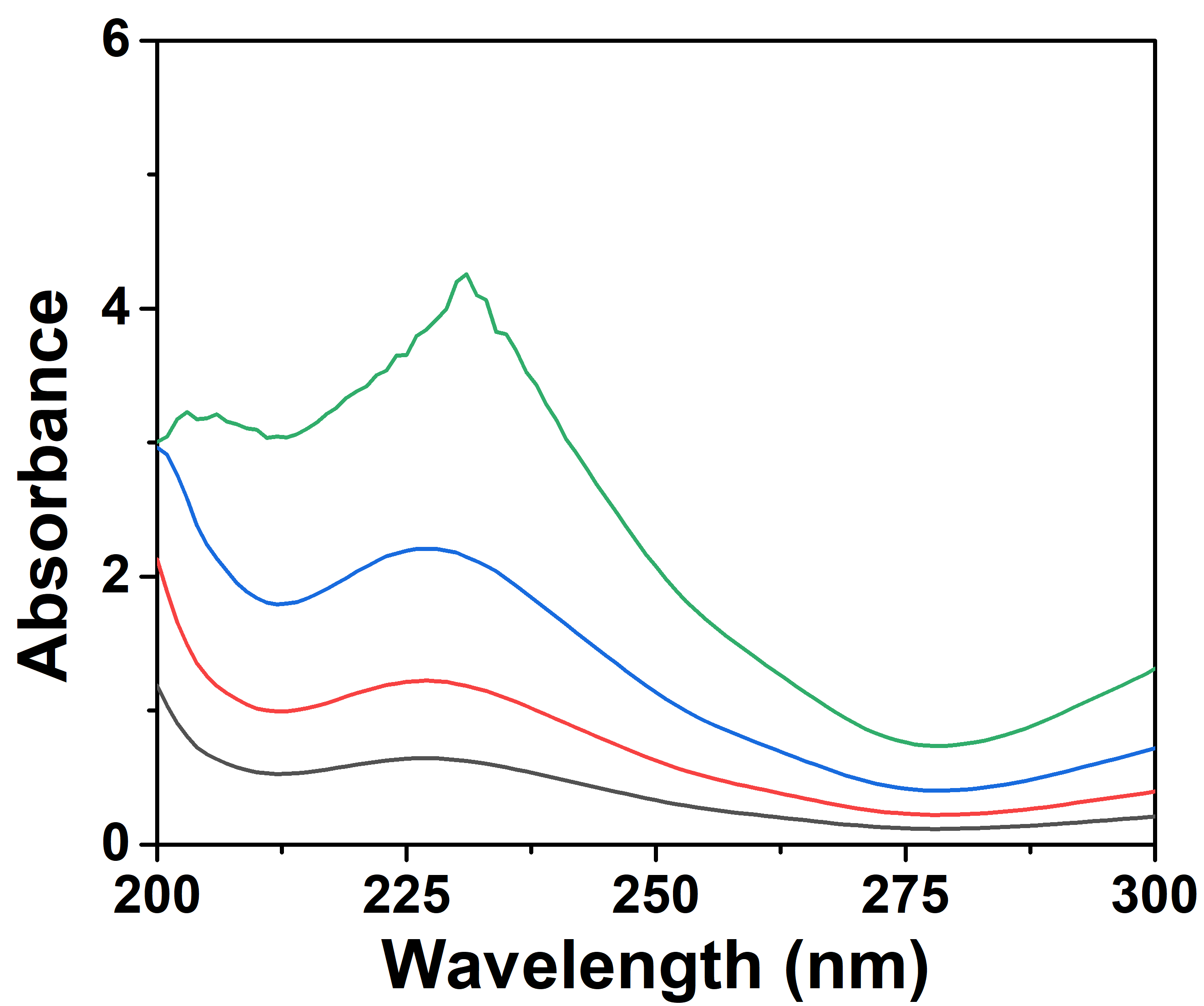


Figure S11. UV standard curve. (Ethanol/Water = 90/10), [nitroaromatic compounds 3] = 0.01565 mg/mL, 0.0315 mg/mL, 0.0625 mg/mL, 0.125 mg/mL

Figure S12. UV standard curve, linear fit. (Ethanol/Water = 90/10), [nitroaromatic compounds 3] = 0.01565 mg/mL, 0.0315 mg/mL, 0.0625 mg/mL, 0.125 mg/mL

Figure S13. UV absorption curve, 5 mg of the main compound, after fully stirring the adsorption of the nitroaromatic compound 3, filtered and rinsed, and dissolved in 10 mL of ethanol. The solution was further diluted 5 times for testing.

**2. Supplementary Tables**

Table S1. Pore structure parameters of various porous organic polymers.

| sample | S_BET_ (m^2^/g) | S_micro_ (m^2^/g) | V_t_ (cm^3^/g) | V_micro_ (cm^3^/g) |
| --- | --- | --- | --- | --- |
| FPOP-TPE | 663 | 392 | 0.41 | 0.16 |
| S-TPE | 419 | 221 | 0.25 | 0.09 |

Table S2. The resulting concentrations are in mg/mL as chart values. Calculation result: adsorption amount per mg of sample Multiply by 10 for the concentration corresponding to the latter. No. 3 nitroaromatic compound 0.7 mg vs 1 mg sample


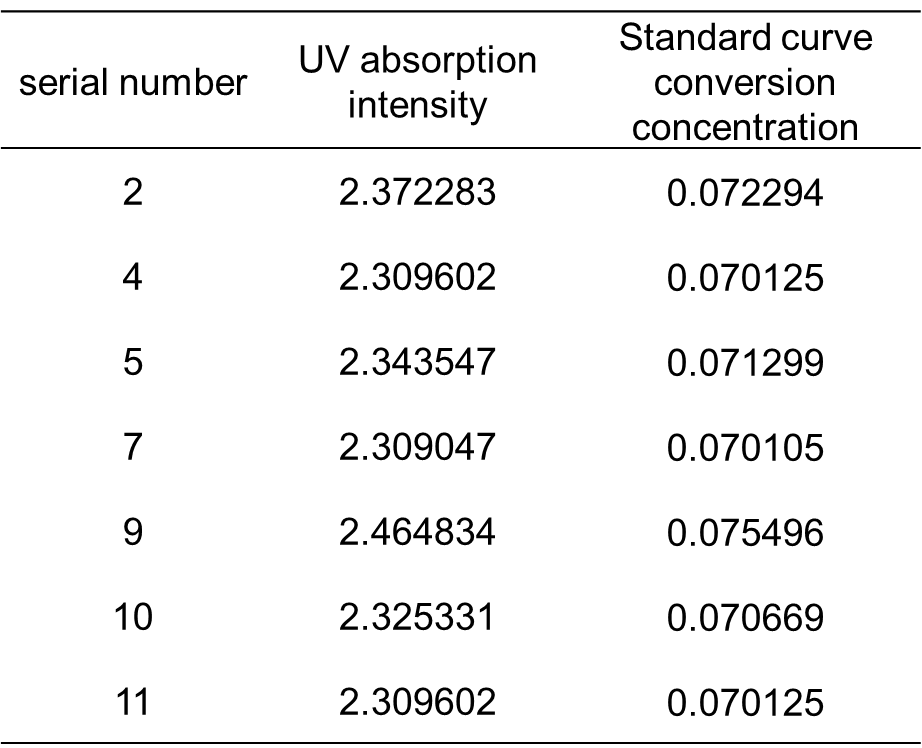


**3. Supplementary Methods**

**Characterization**

**Transmission electron microscopy (TEM) characterizations.** TEM images were obtained using a FEI Tecnai G2 F20 instrument working at 200 kV. Samples were prepared by dip-coating a 500-mesh carbon-coated copper grid from the dilute sample solution allowing the solvent to evaporate.

**Scanning electron microscopy (SEM)** **characterizations.** SEM micrographs were collected with a field emission microscope (Zeiss Ultra 55) with an accelerating voltage of 5.0 kV. Samples were prepared by drop-casting one drop of the dilute sample solution onto a clear silicon wafer and the surface was sputter-coated with gold.

**N_2_ adsorption and desorption experiments.** The BET (Brunauer-Emmett-Teller) surface area, N_2_ sorption isotherms (77K), and pore size distributions were measured using Micromeritics ASAP 2460 surface area and porosity analyzer. Before taking measurements, the samples were degassed for more than 10 h at 120 °C under vacuum (10-5 bar). The BET surface areas and the micropore surface areas were determined by the BET equation and the *t*-plot equation, respectively. The pore size distributions were analyzed by DFT methods via the adsorption branch.

**UV-vis absorption spectra characterizations**. UV-vis absorption spectra were recorded on a Hewlett Packard 8453 UV-Vis spectrophotometer.

**Thermogravimetric analysis (TGA).** TGA were carried out with a TGA5500 at a heating rate of 10 °C /min from 25 to 800 °C in a nitrogen atmosphere.

**Fluorescent spectra characterizations**. Fluorescent emission spectra collected on a Shimadzu RF-5301 fluorophotometer at 298 K.

**Fourier transform infrared (FT-IR) spectra characterizations**. FT-IR were measured within a 4000 to 400 cm^-1^ region on a Bruker TENSOR-27 infrared spectrophotometer (KBr pellet).

**Solid-state NMR characterizations**. ^13^C cross-polarization/magic-angle-spinning (CP/MAS) NMR data were collected on Bruker AVANCE-500 NMR Spectrometer operating at a magnetic field strength of 9.4 T.

**Quantum yield**. The absolute quantum yield was evaluated by Rayleigh scattering using an integrating sphere.

**4. Supplementary References**

1. Li, B.; Guan, Z.; Yang, X.; Wang, W. D.; Wang, W.; Hussain, I.; Song, K.; Tan, B.; Li, T., Multifunctional microporous organic polymers. *J. Mater. Chem. A.* **2014,** *2* (30), 11930-11939.

2. Hou, S.; Razzaque, S.; Tan, B., Effects of synthesis methodology on microporous organic hyper-cross-linked polymers with respect to structural porosity, gas uptake performance and fluorescence properties. *Polym. Chem.* **2019,** *10* (11), 1299-1311.
